# Characterization of the Temperament and Reactivity of Nelore Cattle (*Bos indicus*) Associated with Behavior Scores during Corral Management in the Humid Tropics

**DOI:** 10.3390/ani14121769

**Published:** 2024-06-12

**Authors:** Welligton Conceição da Silva, Jamile Andréa Rodrigues da Silva, Lucieta Guerreiro Martorano, Éder Bruno Rebelo da Silva, Tatiane Silva Belo, Kedson Alessandri Lobo Neves, Raimundo Nonato Colares Camargo Júnior, Cláudio Vieira de Araújo, Luís Gustavo Paixão Vilela, Leonel António Joaquim, Thomaz Cyro Guimarães de Carvalho Rodrigues, José de Brito Lourenço-Júnior

**Affiliations:** 1 Postgraduate Program in Animal Science (PPGCAN), Institute of Veterinary Medicine, Federal University of Para (UFPA), Castanhal 68746-360, Brazil; eder.b.rebelo@gmail.com (É.B.R.d.S.); camargojunior@gmail.com (R.N.C.C.J.); jleokim.a@hotmail.com (L.A.J.); thomazguimaraes@yahoo.com.br (T.C.G.d.C.R.); joselourencojr@yahoo.com.br (J.d.B.L.-J.); 2 Institute of Animal Health and Production, Federal Rural University of the Amazônia (UFRA), Belem 66077-830, Brazil; jamileandrea@yahoo.com.br; 3 Embrapa Eastern Amazon, Santarem 68010-180, Brazil; lucieta.martorano@embrapa.br; 4 Department of Veterinary Medicine, University Center of the Amazon (UNAMA), Santarem 68010-200, Brazil; tatianebelovet@gmail.com; 5 Institute of Engineering and Geosciences, Federal University of Western Pará (UFOPA), Santarem 68040-255, Brazil; kedson_neves@hotmail.com; 6 Institute of Animal Science, Federal University of Western Pará (UFOPA), Santarem 68040-255, Brazil; cvaufmt@gmail.com; 7 Agronomy Department, Lutheran Universities of Brazil (CEULS/ULBRA), Santarem 68025-000, Brazil; vilelagustavo949@gmail.com

**Keywords:** management, containment trunk, temperament score

## Abstract

**Simple Summary:**

The evaluation of reactivity and distress can be used in order to standardize non-invasive indicative methods to evaluate the behavior of animals in the corral, thus developing appropriate management practices that enable the well-being and comfort of animals. The animals were evaluated individually and according to the rearing systems. No significant changes in temperament and reactivity were perceptible when the animals were evaluated between the systems, but changes were perceptible when the animals were evaluated individually. In this case, there was a positive correlation between the physiological variables of rectal temperature (RT) and respiratory rate (RR) and between RT, RR, body surface temperature (BST), and behavioral indicators in corral escape speed (ES), tension score (SS_1), tension score (SS_2), reactivity scale (RS), movement score (MS), and temperament scale (TS).

**Abstract:**

The evaluation of the reactivity and distress of cattle during corral management, by means of subjective scores, aims at the standardization of behavioral indicators, through non-invasive methods, in addition to enabling the development of more appropriate management practices, thus promoting the comfort and well-being of these animals. Therefore, in this study, we aimed to characterize the temperament and distress of cattle managed in a corral using behavioral indicators during the rainiest period. For this, the experiment was conducted on a property located in the municipality of Mojuí dos Campos, during the rainiest quarter (February–April). Thus, 30 male cattle, not castrated, approximately 29 months of age, clinically healthy, and weighing 310 + 20 kg, were divided into three rearing systems: silvopastoral (SP), traditional (SS), and integrated (SI) systems. There were 10 animals per system. Physiological parameters were collected to evaluate rectal temperature (RT) and respiratory rate (RR), as well as body surface temperature (BST), through thermal windows (head and flank infrared temperature and rump infrared temperature). To evaluate temperament and reactivity, scores indicative of corral behavior were used, namely escape speed (ES), tension score (SS_1), tension score (SS_2), reactivity scale (RS), movement score (MS), and temperament scale (TS). The results showed that there was a thermal amplitude of 5.9 °C on average and 8.6 °C at maximum when comparing the structure of the corral and the trees. In addition, the comparisons between the production systems for the behavioral variables did not differ at the 5% significance level, except for ES, where the traditional system differed from the integrated system and the silvopastoral system, showing intermediate average values for both. In addition, there was a positive correlation between the variables RT and RR (r = 0.72; *p* < 0.01), RR and SS_2 (r = 0.38; *p* = 0.04), flank infrared temperature and MS (r = 0.47; *p* = 0.01), rump infrared temperature and RS (r = 0.37; *p* = 0.04), SS_1 and RS (r = 0.41; *p* = 0.02), SS_1 and SS_2 (r = 0.39; *p* = 0.03), RS and SS_2 (r = 0.58; *p* = 0.00), RS and MS (r = 0.50; *p* = 0.01), RS and TS (r = 0.61; *p* = 0.00), SS_2 and MS (r = 0.51; *p* = 0.00), SS_2 and TS (r = 0.47; *p* = 0.01), and MS and TS (r = 0.44; *p* = 0.02), and a negative correlation between ES and TS (r = −0.42; *p* = 0.02). The rainy season had a major influence on the evaluation of temperature and distress levels during handling in the corral, as evidenced by the association between physiological and behavioral parameters.

## 1. Introduction

Brazil is considered the world’s largest beef exporter, occupying an important position in cattle farming [1]. In recent years, there has been greater concern on the part of livestock farmers about animal welfare, i.e., how these animals are being treated on farms and the possible impacts on production and consequently on the quality of the final product [2,3,4,5].

Livestock farming is significant to the economy and social aspects of the eastern Amazon, due to its natural pasture areas with great biodiversity, making this region crucial for developing this productive sector more and more. Considering environmental and climatic factors, as cattle are sensitive to climatic variations, they can present health and welfare problems commonly observed when they are exposed to high temperatures [6,7,8,9,10]; this exposure to high temperatures impacts the temperament and distress of the animals [11].

Temperament can generally be defined as “good” or “bad”, depending on the presence or absence of desirable characteristics [12,13,14,15]. These characteristics affect the performance and well-being of the animals, so many farms have started to invest in alternatives aimed at facilitating management practices in order to minimize distress for both animals and humans, especially losses caused by distress during the journey to slaughter [16,17].

In general, temperament is defined as the variation between animals and their reaction to a given stimulus; it also refers to inborn hereditary traits rather than acquired traits [18,19,20]. Thus, alternatives that seek to assess temperament through behavioral analyses in management situations are becoming increasingly common in cattle farming, given that temperament is a set of behaviors, mainly of animals in relation to humans [21,22,23], but it also occurs in relation to the environment.

There are various temperament indicators used in scientific research, due to their usefulness and ease of application. In general, the methodologies are classified according to the condition in which they will be tested, varying between tests with and without restraint [24,25,26,27,28]. The use of the escape velocity score is used to assess temperament through the time it takes them to cover the distance between the exit point and an open area. In terms of temperament, animals are assessed using scores ranging from one to four in relation to the environment and human presence [29].

Other methods, such as the tension score, reactivity scale, and movement score, are used to assess the behavior of animals in situations of restricted space and proximity to humans, which is related to fear in general. Thus, research aimed at evaluating the behavioral and physiological responses of cattle associated with the environment in which they are raised and the interaction between them and humans during handling, helps to standardize indicators, reduce operating costs, and produce better quality products. Therefore, this study aimed to characterize the temperament and distress of cattle managed in a corral using behavioral indicators during the rainiest period.

## 2. Materials and Methods

### 2.1. Experiment Site and Climate Characterization

**Figure 1:**
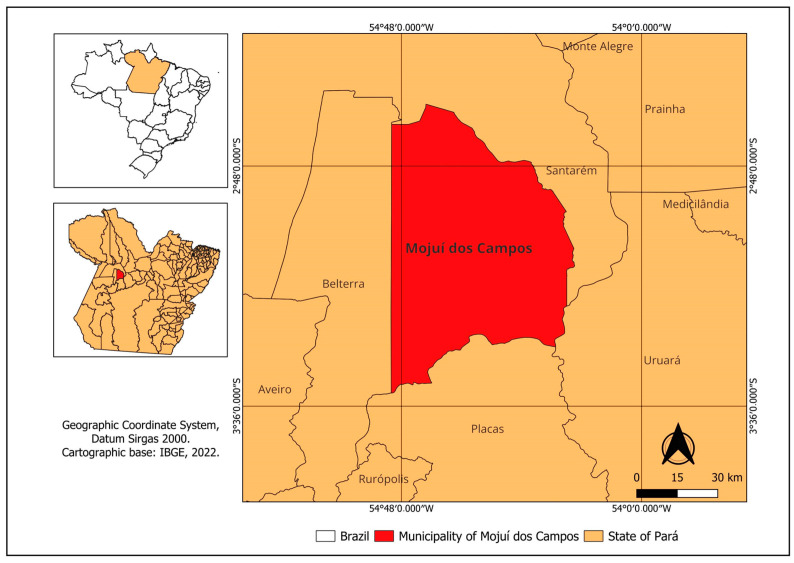
Map of the study site.

The experiment was carried out on a rural property in the municipality of Mojuí dos Campos, Pará, (Figure 1) during the wettest months (December to May). According to Martorano et al. [30], the climate in the region is hot and humid with an average temperature of 25.6 °C and air humidity ranging from 84 to 86%. Furthermore, the periods of the greatest and least intensity of rainfall in this region can be divided into quarters, with the wettest being observed between the months of February and April [31].

### 2.2. Experimental Animals, Characterization of the Breeding System, and Management in Pens

Thirty uncastrated male Nelore cattle, aged approximately 24 months old and clinically healthy with an average weight of 310 ± 20 kg, were used in the study. They were divided into three rearing systems identified as follows: integrated (IS), traditional (SS), and silvopastoral (SP) systems.

The animals in the integrated system (IS) group were placed in a paddock with 20% of the area shaded by native trees, and had access to drinking water (*ad libitum*) and a bathing area. In the silvopastoral system (SP), the cattle remained in paddocks with the same characteristics as the IS group, but without access to a bathing area. Finally, in the traditional system (SS), the animals stayed in the paddock without trees or shading elements, and also without access to a bathing area, but with access to drinking water (*ad libitum*). The total experimental area was 15.3 ha of *Brachiaria brizantha* cv. Marandú, divided into nine 1.7 ha paddocks, three per treatment, considered suitable for conditioning the animals.

The environmental and climatic conditions were the same for each experimental group of animals. The cattle were walked to the waiting pen in a calm and quiet manner, with each treatment being taken in turn, avoiding distress during this period. The animals underwent an adaptation period for handling in the corral, lasting seven days. They were led into the corral slowly, on foot, without shouting or the presence of humans or other animals to avoid distress. Thus, every 28 days the animals were handled in a containment trunk for weighing and the evaluation of physiological parameters such as respiratory rate (RR) and rectal temperature (RT).

**Figure 2:**
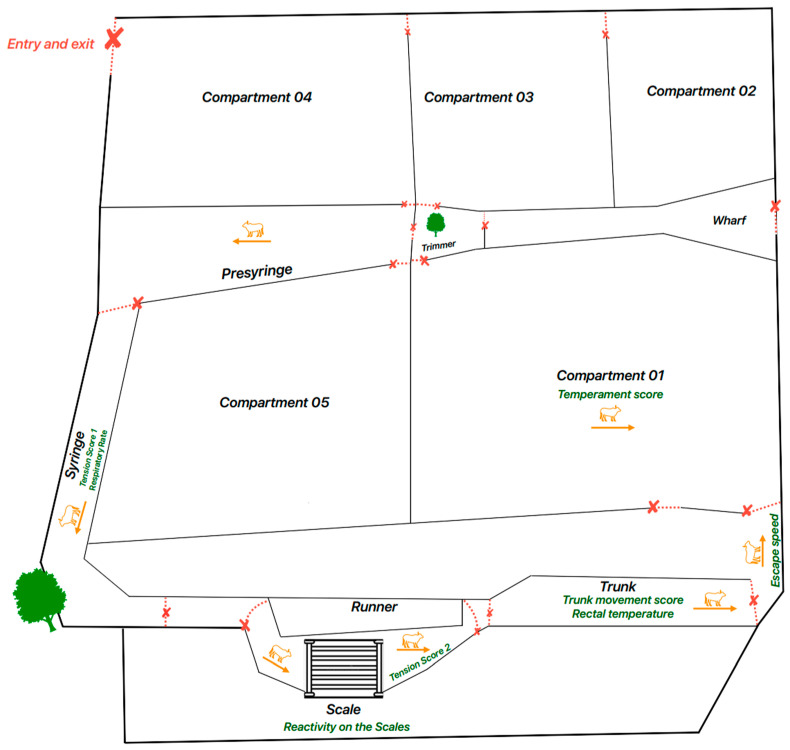
Infographic of the corral used to handle the animals and the respective places where the parameters were evaluated. X in red indicates the areas of the corral where data were collected according to the parameter.

The corral has three waiting compartments with elements that simulate the farming system in which they were kept, such as trees and a hose bath (Figure 2), as well as a sunbathing area for the SS group.

### 2.3. Evaluation of Temperament and Reactivity

Evaluations were carried out by observation using methodologies to assess temperament, tension, trunk movement, reactivity on the scales, and escape speed. Records were taken every 28 days, during weighing, in the rainiest period between February and April.

Initially, the animals were led to the syringe for the tension score (SS_1), followed by weighing on the scale and evaluation of the reactivity scale (RS), after which they entered the corridor for the tension score (SS_2). Next, a containment trunk was used for the movement score (MS) and blood sampling. Then, the animals were allowed to exit from the trunk to assess the escape speed (ES), after which the animals remained in one of the corral compartments for the temperament scale (TS) assessment.

### 2.4. Tension Score (SS_1 and SS_2)

The tension score is related to the general tension of each animal’s body. It aims to assess muscle tone and the movement of the head, neck, and tail more closely; the scores that were assigned are shown in Table 1. Two tension scores were given for the syringe corridor (SS_1) and the corridor between the scales and the trunk (SS_2) [21].

### 2.5. Scale Reactivity Score (RS)

The reactivity score aims to assess the behavior of cattle while in a restraint, so that scores which measure the animal’s movements can be assigned (Table 1) [21]. It is carried out in the first four seconds of observation, after the animal enters the scales, by an observer who checks the scores that consider the following behaviors: movement, audible breathing, posture, mooing, and kicking [32].

### 2.6. Movement Score (MS)

The movement score [21] evaluates the movement of cattle in the containment trunk, with little or no space for 4 s, to assign scores (Table 1).

### 2.7. Escape Speed (ES)

Escape velocity is defined as the speed at which the animal leaves the trunk. It is assessed using a stopwatch to measure the time in seconds that it takes for the animal to leave the trunk towards an open place over a given distance, which is then converted into exit velocity (m.s^−1^), with the slowest animals being those of “good” temperament [13]. The evaluation takes place in three classes (Table 1).

### 2.8. Temperament Score (TS)

The temperament score is a method used in genetic improvement in agriculture, aimed at evaluating the reactions of animals inside the pen, after leaving and handling in the trunk, considering the scores in Table 1 [19].

**Table 1:** Animal tension evaluation ethogram (SS_1 and SS_2), trunk movement evaluation score (MS), escape velocity ethogram (ES), temperament score (TS), and reactivity in the bucket (RS).

**Tension Score (SS_1 and SS_2)**		
**Score**	**Rating**	**Definition**
1	Relaxed	The animal has no sudden movements of the tail, head, and neck, with regular muscle tone;
2	Alert	The animal shows few sudden movements of the tail, head, and neck;
3	Tense	The animal shows continuous and vigorous movements of the tail, head, strength, and flight;
4	Very tense	The animal is paralyzed or has a “freeze” reaction and visible muscle tremors.
**Trunk Movement Score (MS)**		
**Score**	**Classification**	**Definition**
1	No movement	Relaxed animal with no sudden movements;
2	Little movement	Slightly restless, alert animal;
3	Frequent movement	Shows vigorous movements, tries to escape, alert;
4	Constant and vigorous movement	Very tense, panting, animal jumps and struggles. May have a visible sclerotic membrane and muscle tremors.
**Escape Speed (ES)**		
**Score**	**Classification**	**Definition**
1	Slow animals	More than one deviation below the mean;
2	Medium animals	The average is equal to the deviation;
3	Fast animals	One deviation above the mean.
**Temperament Score (TS)**		
**Score**	**Classification**	**Definition**
1	Docile animals	Animal walks slowly, is not bothered by the environment and allows proximity;
2	Attentive animals	Trotting or running for a few seconds without aggression and allows moderate proximity;
3	Agitated animals	Runs for the duration of the observation, seeking escape and without proximity;
4	Aggressive animals	Annoyed by the environment and human presence throughout the assessment, running and jumping against fences and obstacles.
**Reactivity on the Scales (RS)**		
**Score**		**Classification**
1		Non-reactive animal
2		Slightly reactive animal
3		Reactive animal
4		Very reactive animal

Note: Adapted from Fordyce et al. [21] and Burrow et al. [33].

### 2.9. Body Surface Temperature (BST) Analysis

**Figure 3:**
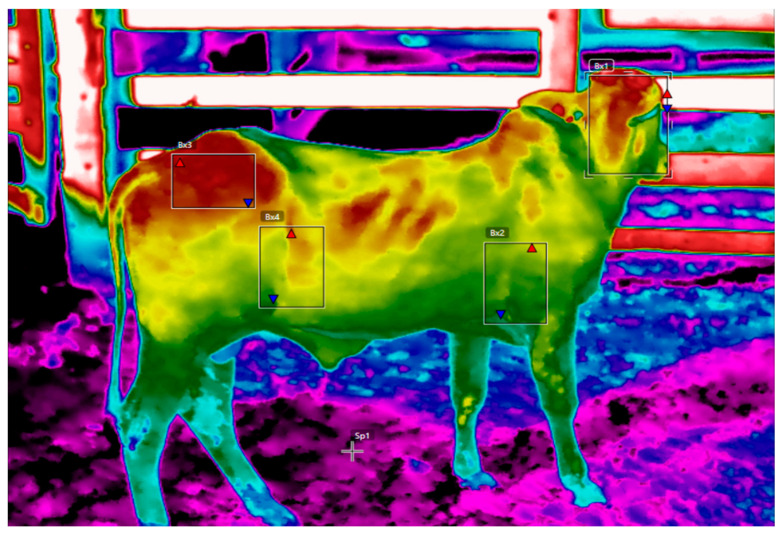
Thermal windows where thermal targets were imaged in the cattle studied. B × 1 = head, B × 2 = armpit, B × 3 = flank infrared temperature, and B × 4 = rump infrared temperature. Red and blue arrows indicate the boundaries of the thermal windows.

The BST was analyzed using an infrared thermometer with a scientific thermographic camera during the rainiest period (February/March/April). The evaluations were carried out at the time of collection each month between 12:00 and 15:00, specifically in the anatomical areas of the head, the armpit, the flank, and the rump, using 310 imaged points (Figure 3). Data were collected using a scientific IRT camera, with an emissivity set at 0.95, a fixed 25 mm objective, a temperature range of −40 to 150 °C, a sensitivity of 50 mk (>0.05 °C at an ambient temperature of 30 °C), and a spectral range of 0.7 to 100 μm; however, the targets photographed have a response between 0.7 and 3.0 μm, with an optical resolution of 640 × 480 pixels and a maximum emissivity index of 0.9. The temperature and humidity were controlled by positioning the observer against the sun, so as not to interfere with the camera. The thermograms were analyzed using Flir Tools software, version 6.3, with the Rainbow HC palette selected. The operator used the camera at eye level, always maintaining coherence and without the aid of a tripod, focusing perpendicular to the target, following the methodological guidelines proposed by Silva et al. [3] and Silva et al. [5].

The structure of the corral, specifically the fence, was also assessed. In general, the thermograms of the cattle were acquired inside the corral, in the environment, and with the animal standing. The systems adopted thermograms that were acquired at an orthogonal distance of 5 m, outside the bull’s flight distance [34], to avoid distress and reassure the cattle.

### 2.10. Statistical Analysis

Initially, the variables evaluated were allocated into two groups. The first group was made up of physiological characteristics: RT, RR, head, armpit, flank infrared temperature, and rump infrared temperature. A second group was made up of characteristics related to animal behavior: SS_1, RS, SS_2, MS, TS, and ES.

For the statistical analyses, first, the multivariate analysis of variance (Manova) technique was performed on group 1, using Roy’s greatest root test, in order to detect significant differences between the vectors of production system means. In this situation, the multivariate linear model was represented by the following:

Y = Xβ + ε

where Y is the matrix with n observations in *p* characteristics of the ith group; X is the matrix of incidence of the fixed effects of production systems related to each observation in Y; β is the vector of solutions of production systems in *p* characteristics; and ε is the vector of residues of the observations of the *p* characteristics in Y.

In the next step, depending on the statistical significance of the mean vectors, the multivariate technique of Fischer’s linear discriminant analysis was applied, using the canonical variables technique.

The dimensionality of the parametric space in this group was obtained by testing the null hypothesis in which the canonical correlation obtained through each eigenvalue was considered to be equal to zero. Based on the relevance of each canonical variable, the dispersions of treatments were obtained in relation to the canonical treatment means obtained using the two main canonical variables, in order to discriminate the differences between production systems.

The confidence region of the canonical means was obtained using the radius disk as follows:

$$\left\{{\langle \frac{x_{\left(t,\alpha \right)}^{2}}{n}\rangle }^{0.5}\right\}$$

where *t* represents the number of treatments and *x*^2^ is the chi-squared distribution for the level of significance (*p* = 0.05) with sample size *n*.

Once we had the two canonical variables for the original variables in group 1, we compared the production systems by means of the canonical means using Tukey’s test.

For the variables in group 2, in order to test possible differences between production systems, the non-parametric Kruskal Wallis test was applied for the SS_1, RS, SS_2, MS, and TS variables, and the F test was applied for the ES variable, the latter of which was transformed to the logarithmic scale in order to bring it closer to the normal distribution.

In a further step, Pearson’s linear correlation was obtained between the variables in group 2 and the canonical variables. All statistical analyses were carried out using the Statistical Analysis System [35] on Demand for Academics program, adopting a significance level of 0.05.

## 3. Results

**Figure 4:**
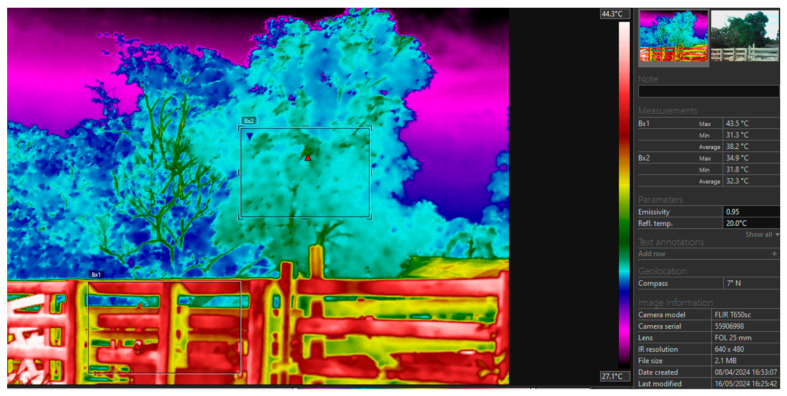
Thermographic images of the corral area where the animals were handled. B × 1 = Tree temperature; B × 2 = Corral temperature.

The corral environment had wooden fences with an average temperature of 38.2 °C and a maximum of 43.5 °C. The presence of trees in the corral had a temperature of 32.3 °C and a maximum of 34.9 °C, i.e., a temperature range of 5.9 °C on average and 8.6 °C at maximum when comparing the structure of the corral and the trees (Figure 4).

The result of the multivariate Roy’s greatest root test was 28.81 with an associated probability of less than 0.0001, indicating differences between the vectors of the average estimates between the production systems. Based on the values of the confidence intervals of the average estimates (Table 2), it can be said that, for TR, the silvopastoral system had a lower estimate than the traditional system, with the silvopastoral system having an intermediate value. For the FR variable, the silvopastoral and integrated systems had averages in the same ranges and lower than those observed in the traditional system. The average estimate for head temperature in the integrated system was higher than that of the other two systems and they had averages in the same ranges.

The results of the discriminant analysis between production systems using the first two canonical variables (Table 3) indicate that the first canonical variable explained 76.4% of the total variation contained in the original physiological variables, with RT, followed by rump infrared temperature, flank infrared temperature, and head temperature, having the highest weighting in this canonical variable. In the second canonical variable, which explained 23.6% of the total variation contained in the original variables, the head and flank infrared temperature variables were weighted more heavily.

The estimates of canonical means and their respective standard deviations, by production system, are shown in Table 4. For the discrimination between systems obtained using the Tukey test, in the first and most relevant canonical variable, the three systems differed from each other, with higher and lower estimates for the traditional and silvopastoral systems, respectively. Thus, higher RT and BST values are expected in the head, flank infrared temperature, and rump infrared temperature regions in the traditional system. In the second canonical variable, due to the greater influence of head temperature weighting, the integrated system had a higher canonical mean than the other two systems.

**Figure 5:**
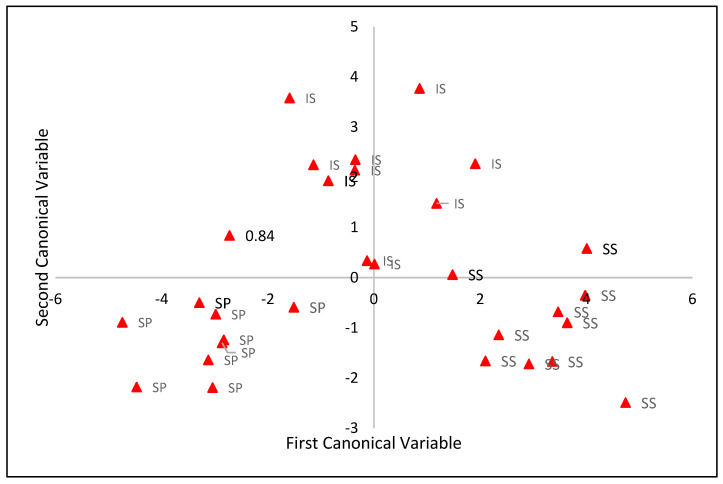
Dispersion between the canonical values of the integrated (IS), silvopastoral (SP), and traditional (SS) production systems, considering the two canonical variables.

**Figure 6:**
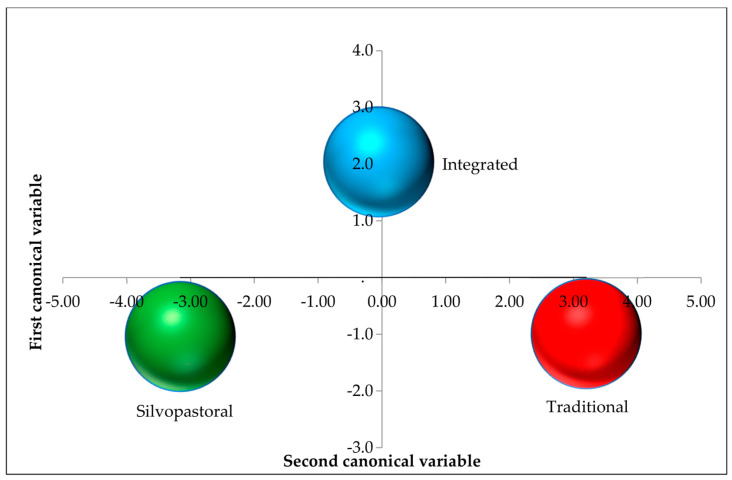
Dispersion between the mean canonical values between the integrated system, silvopastoral, and traditional production systems, considering the two canonical variables.

Figure 5 and Figure 6 show the dispersions of the production systems in relation to the canonical values and averages, respectively, indicating the discrimination between the production systems in the set of two canonical variables. According to the 95% confidence radius of the canonical averages, the three systems differ from each other in the set of two canonical variables (Figure 2).

Comparisons between production systems for behavioral variables did not differ at the 5% significance level, except for the logarithm of ES, where the traditional system differed from the integrated system and the silvopastoral system showed average values intermediate to both (Table 5).

Pearson’s correlation coefficients between the two canonical variables and the behavioral variables (Table 6) indicated that, with the exception of TS with Vc2, all the other correlations were not significant.

Table 7 shows the correlation data of the physiological and behavioral variables, in which there was a positive correlation between the variables RT and RR (r = 0.72; *p* < 0.01), RR and SS_2 (r = 0.38; *p* = 0.04), flank infrared temperature and MS (r = 0.47; *p* = 0.01), rump infrared temperature and RS (r = 0. 37; *p* = 0.04), SS_1 and RS (r = 0.41; *p* = 0.02), SS_1 and SS_2 (r = 0.39; *p* = 0.03), RS and SS_2 (r = 0.58; *p* = 0.00), RS and MS (r = 0. 50; *p* = 0.01), RS and TS (r = 0.61; *p* = 0.00), SS_2 and MS (r = 0.51; *p* = 0.00), SS_2 and TS (r = 0.47; *p* = 0.01), and MS and TS (r = 0.44; *p* = 0.02). There was a negative correlation between ES and TS (r = −0.42; *p* = 0.02).

## 4. Discussion

By analyzing the mean RT and RR between the traditional system and silvopastoral system groups, it was possible to perceive a reduction than when compared to the integrated system, thus suggesting that the presence of shading can benefit the animals due to the reduction of direct exposure to sunlight, positively impacting their physiological performance [2,5,34,36,37].

Temperatures are higher in the traditional system due to the lack of trees that can provide shade to the animals when compared to the silvopastoral system. Environments without the availability of trees favor the exacerbated exposure of the animal to solar radiation and consequently the heating of the soil, which also contributes to the increase in the animal’s body temperature [38,39,40]. The integrated system, which presents both tree shade and a bathing area, have intermediate temperature values between the two systems previously mentioned [4,41].

The presence of a bathing area contributes to the process of regulating body temperature through the evaporation of water, making the environment more conducive to the animal, acting together with the shading of trees to ensure thermal comfort to the animal. In the traditional system, it was possible to observe higher RR values due to the heat distress to which they are subject, such as a lack of shading and a bathing area; on the other hand, the silvopastoral system and integrated system present RR values within the normal range, with the only distress time observed at 6:00 pm [3,42,43,44].

Physiologically, it can be observed that the animals raised in the traditional system are unable to carry out their heat dissipation mechanisms. Also, when these animals are exposed to high temperatures, the thermoregulation process begins, through the increase of RR and RT [45,46]. RR is a mechanism that attempts to regulate body temperature through water evaporation, which can also be indicative of heat distress in these animals [47,48].

On the other hand, in systems with the availability of shade and a bathing area, it is possible to observe body temperature values within the reference values, due to the heat dissipation mechanism being more efficient, due to the reduction of the ambient temperature, resulting in lower RR values within the normal range [49,50]. In addition, the absence of shade can lead to direct sunlight exposure of the soil and high temperatures, further contributing to the increase in body temperature, causing heat distress [51,52,53].

RR is an indicator of well-being and can increase in situations of adaptability due to the increase in temperature and distress of the animal. RR values are reported above the basal rate at 60 respiratory incursions per minute, for each degree above the temperature of 21.3 °C [54,55,56]. On the other hand, in the evaluation of BST, it was evidenced that the head region presented a higher temperature when compared to other regions of the body, due to the greater blood supply in this region [57,58,59].

Higher values of physiological variables such as RR and body (surface) temperature in the canonical evaluation may indicate the impacts of the production environment on animal health. In addition, these impacts may reflect a reduction in nutrients, environmental humidity, and distress caused by inappropriate practices [60,61,62].

The values obtained in the second canonical variable are attributed to the variables in relation to head and flank infrared temperature, suggesting that these have a lower weight when compared to RT to differentiate the impacts of the environment. Factors such as wind and time of exposure to sunlight can influence the temperature variation [4,63]. High RT and BST values in the head and flank infrared temperature and rump infrared temperature regions in the traditional system explain the relationship between exposure to direct solar radiation and the physiological mechanism of heat dissipation [64,65].

The different values obtained in ES between the traditional system and integrated system indicate variations related to the behavior and physiology of the animal in response to the environment in which it is inserted [2,66]. In addition, ES is considered an indicator for the well-being and comfort of animals in open environments, but with limitations such as the corral, and may also indicate a stimulus in the search for alternatives to reduce body temperature [67,68,69].

In the evaluation of the two canonical variables, it was characterized that the animals did not present different behaviors when comparing the rearing systems with the exception of TS with Vc2. This suggests that, in general, there were no perceptible and substantial differences in distress and discomfort between the environments, characterizing a process of adaptation of the animals even in different environments, which is a common characteristic of the breed in this study [70,71,72].

On the other hand, the positive correlation between RT and RR suggests that there is a relationship between physiological variables; as RT increases, RR will also increase, suggesting a coordinated response by the body as a way of trying to regulate body temperature [65,73,74].

In addition, significant correlations observed, such as between RR and SS_2, may indicate that the increase in RR is associated with the degree of tension or distress of the animal during management, in the same way that the correlation between RS and MS (r = 0.50; *p* = 0.01) may indicate aspects of the animal’s behavior due to environmental or management stimuli [45,51,75,76].

The positive correlation between the flank infrared temperature variable and the MS may characterize more active or restless animals with more pronounced movements. Alternatively, increased movement in the trunk can be an indication of the animals’ discomfort, increasing their behavioral responses to express their discomfort due to limited space and human presence [53,73,77].

The positive correlation between the variable rump infrared temperature and RS can be characterized as a greater movement of the rump infrared temperature region as a form of reactivity during weighing, also indicating discomfort due to limited space, which can be interpreted as a discomfort response, where more reactive animals express their distress through more pronounced movements in the rump infrared temperature region [40,74,78,79].

The positive correlation between SS_1 and RS indicates that when there is greater tension in the corridor of the pen, the animals tend to be more reactive on the scales, while factors such as space, noise, and the presence of other animals can also influence their reactivity during handling [52,59,70,80].

The positive correlation between SS_1 and SS_2 may indicate a consistency in the perception of tension along the corridor of the corral during different handling sessions. This suggests that the perceived level of tension in the corral aisle is relatively stable over time or between different handling occasions. This constancy in the perception of distress may have significant implications for animal welfare, as it suggests that certain characteristics of the pen aisle environment may exert a persistent influence on the emotional state of animals during handling [76,81,82].

The positive correlation between RS and SS_2 indicates that the greater the tension of the animals in the corridor, the greater the reactivity on the scale. This suggests that the level of tension in the pen may have a direct influence on the animals’ emotional response during handling. Similarly, the positive correlation between RS and MS suggests that reactive animals move more within the trunk, indicating greater reactivity and restlessness or discomfort due to limited space [19,76,83,84].

In addition, the positive correlation between RS and TS highlights the influence of the individual temperament of the animals on their response during weighing. This suggests that more reactive animals tend to show a greater emotional response and a more aggressive temperament during weighing, which can affect their tranquility and well-being during handling [85,86,87].

The positive correlation between SS_2 and TS suggests that animals that are more tense during procedures such as weighing tend to have a more reactive or nervous temperament, which is reflected individually in their responses to distress or the environment. Thus, more reactive animals may show more pronounced signs of tension in handling situations [88,89,90].

Similarly, the positive correlation between MS and TS indicates an individual distress temperament of the animals, with more reactive animals being more likely to move more during situations of distress or discomfort [91,92,93,94].

A larger number of cattle can be used in future experiments and other variables can be adopted in order to maximize the understanding of these animals in each production system in the Brazilian Amazon.

## 5. Conclusions

The rainy season had a great influence on the evaluation of temperature and distress levels during management in the corral, as evidenced by the relationship between environmental stimuli and the physiological and behavioral reactions of the animals. Physiological variables such as TR, FR, and BST in the flank infrared temperature, rump infrared temperature, and other thermal windows showed significant variations and higher levels of change. As a result, the traditional system showed higher TR and BST, especially in the head and flank infrared temperature and rump infrared temperature regions, indicating higher levels in these regions during confinement, with these temperatures being relevant in differentiating the production systems and in characterizing the distress and temperament of the animals. The silvopastoral and integrated systems were more efficient in terms of cattle welfare than the traditional system. It was noted that the correlations were considered to be good because they portrayed the temperament of the animals in a coherent way, indicating that during the time spent in corral management, the measurement of physiological and behavioral variables should be used to measure the temperament of the cattle.

## Figures and Tables

**Table 2:** Estimated means and standard deviations for the physiological variables measured in the animals in each production system.

Continuous Variables	Integrated			
	Average	SD	LL	UL
RT	37.86	0.38	37.58	38.13
RR	28.53	1.68	27.32	29.73
Head	42.31	0.65	41.84	42.79
Axilla	40.88	0.26	40.7	41.07
Flank infrared temperature	40.49	0.23	40.32	40.66
Rump infrared temperature	43.56	0.22	43.4	43.72
	**Traditional**			
	**Average**	**SD**	**LL**	**UL**
RT	38.31	0.28	38.1	38.51
RR	35.2	2	33.76	36.63
Head	41.21	0.48	40.86	41.55
Axilla	41.05	0.34	40.8	41.3
Flank infrared temperature	40.29	0.17	40.17	40.42
Rump infrared temperature	43.46	0.25	43.27	43.65
	**Silvopastoral**			
	**Average**	**SD**	**LL**	**UL**
RT	37.38	0.31	37.15	37.6
RR	27.46	1.56	26.34	28.58
Head	40.71	0.45	40.38	41.03
Axilla	40.91	0.23	40.74	41.08
Flank infrared temperature	40.44	0.31	40.22	40.67
Rump infrared temperature	43.48	0.19	43.34	43.62

Note: RT = rectal temperature; RR = respiratory rate; SD = standard deviation; LL = lower limit; UL = upper limit.

**Table 3:** Estimates of the weighting coefficients of the first (Vc1) and second (Vc2) canonical variables, estimates of eigenvalues, proportion explained by the canonical variable (proportion), and F test value and its probability (*p*-value) for the test of relevance of the canonical variable.

Continuous Variables	Weighting Coefficients	
	Vc1	Vc2
RT	1.992	0.992
RR	0.481	−0.185
Head	1.149	1.688
Armpit	−0.274	−0.927
Flank infrared temperature	−1.510	1.031
Rump infrared temperature	−1.757	0.299
Eigenvalue	7.515	2.319
Proportion	0.764	0.236
F-value	15.83	10.67
*p*-value	<0.01	<0.01

Note: RT = rectal temperature; RR = respiratory rate; Vc1 = canonical variable 1; Vc2 = canonical variable 2.

**Table 4:** Estimates of canonical means with their standard deviations for the first two canonical variables referring to the set of variables in each production system.

Production System	Canonical Averages	
	Vc1	Vc2
	Average ± Standard Deviation	Average ± Standard Deviation
Integrated	−0.047 ± 1.090 B	2.043 ± 1.149 A
Silvopastoral	−3.161 ± 0.907 C	−1.044 ± 0.900 B
Traditional	3.208 ± 0.993 A	−0.998 ± 0.931 B

Note: Vc1 = canonical variable 1; Vc2 = canonical variable 2. Different letters in the lines indicate statistical difference (*p* < 0.05).

**Table 5:** Means and standard deviations for the categorical behavioral variables and continuous physiological variables with the Kruskal Wallis test values (for categorical) and F test (for continuous) with their probability of significance values (*p*-value), according to the production system.

**Categorical Variables**	**Parameters**	**Systems**			**Kruskal Wallis**
		**Integrated**	**Silvopastoral**	**Traditional**	**(*p*-Value)**
SS_1	Average	1.30	1.46	1.30	2.01 (0.3653)
	SD	0.42	0.32	0.24	
	Average				
RS	SD	1.63	1.3	1.26	0.45 (0.7970)
	Average	0.8	0.24	0.21	
	SD				
SS_2	Average	1.86	1.63	1.43	3.61 (0.1642)
	SD	0.59	0.42	0.38	
	Average				
MS	SD	1.83	1.33	1.16	2.09 (0.3501)
	Average	1.04	0.35	0.23	
	SD				
TS	Average	1.5	1.06	1.06	5.54 (0.0624)
	SD	0.59	0.21	0.14	
**Variables Continuous**	**Parameters**	**Systems**			**F test**
		**Integrated**	**Silvopastoral**	**Traditional**	**(*p*-Value)**
ES	Average	7.5	11.86	9.66	2.83 (0.0765)
	SD	3.93	5.28	2.66	
Log(ES)	Average	1.91 B	2.38 A	2.23 AB	3.59 (0.0415)
	SD	0.44	0.45	0.27	

Note: RT = rectal temperature; RR = respiratory rate; SD = standard deviation; ES = Escape speed; SS_1 = tension score; SS_2 = tension score; RS= reactivity scale; MS = movement score; TS = temperament scale.

**Table 6:** Pearson’s correlation coefficients, with probability associated with the null hypothesis of the null correlation of the canonical variables with the behavioral variables.

Variable		Canonical Variable	
Behavioral		Vc1	Vc2
SS_1	Correlation	0.175	−0.258
	*p*-value	0.35	0.16
RS	Correlation	0.005	0.231
	*p*-value	0.97	0.21
	Correlation		
SS_2	*p*-value	0.109	0.234
	Correlation	0.56	0.21
	*p*-value		
MS	Correlation	0.104	0.263
	*p*-value	0.58	0.15
	Correlation		
TS	*p*-value	0.009	0.457
	Correlation	0.96	0.01
	*p*-value		
ES	Correlation	0.144	−0.175
	*p*-value	0.44	0.35

Note: RT = rectal temperature; RR = respiratory rate; SD = standard deviation; ES = escape speed; SS_1 = tension score; SS_2 = tension score; RS = reactivity scale; MS = movement score; TS = temperament scale. Vc1 = canonical variable 1; Vc2 = canonical variable 2.

**Table 7:** Pearson’s linear correlation coefficient between behavioral and physiological variables with probability of significance values (*p*-value).

Variable	Description	RR	Head	Axila	Flank	Rump	ES	SS_1	RS	SS_2	MS	TS
RT	r	0.72	0.06	0.14	0.06	0.15	0.02	0.22	−0.11	−0.23	−0.27	0.09
	*p*-value	<0.01	0.77	0.47	0.76	0.43	0.90	0.25	0.56	0.22	0.16	0.64
RR	r		−0.15	0.24	−0.22	0.01	0.05	0.32	−0.12	0.38	0.14	−0.13
	*p*-value		0.42	0.20	0.23	0.97	0.79	0.08	0.52	0.04	0.45	0.50
Head	r			0.02	0.04	0.11	−0.17	−0.06	0.35	0.33	0.16	−0.11
	*p*-value			0.90	0.85	0.56	0.37	0.75	0.06	0.07	0.39	0.55
Armpit	r				0.11	0.33	0.03	0.05	0.31	0.20	0.11	0.17
	*p*-value				0.56	0.08	0.88	0.78	0.10	0.28	0.56	0.38
Flank infrared temperature	r					0.03	0.13	0.03	0.22	0.06	0.47	−0.21
	*p*-value					0.89	0.51	0.88	0.23	0.77	0.01	0.28
Rump infrared temperature	r						0.08	−0.18	0.37	−0.08	0.25	0.00
	*p*-value						0.66	0.33	0.04	0.66	0.19	0.98
ES	r							−0.17	−0.34	−0.34	−0.31	−0.42
	*p*-value							0.36	0.06	0.07	0.10	0.02
SS_1	r								0.41	0.39	0.22	0.10
	*p*-value								0.02	0.03	0.25	0.59
RS	r									0.58	0.50	0.61
	*p*-value									0.00	0.01	0.00
SS_2	r									1.00	0.51	0.47
	*p*-value										0.00	0.01
MS	r											0.44
	*p*-value											0.02

Note: RT = rectal temperature; RR = respiratory rate; ES = escape speed; SS_1 = tension score; SS_2 = tension score; RS = reactivity scale; MS = movement score; TS = temperament scale.

## Data Availability

The data presented in this study are available upon reasonable request from the corresponding author.
